# The sequence context in poly-alanine regions: structure, function and conservation

**DOI:** 10.1093/bioinformatics/btac610

**Published:** 2022-09-15

**Authors:** Pablo Mier, Carlos A Elena-Real, Juan Cortés, Pau Bernadó, Miguel A Andrade-Navarro

**Affiliations:** Faculty of Biology, Institute of Organismic and Molecular Evolution, Johannes Gutenberg University Mainz, 55128 Mainz, Germany; Centre de Biologie Structurale (CBS), Université de Montpellier, INSERM, CNRS, 34090 Montpellier, France; LAAS-CNRS, Université de Toulouse, CNRS, Toulouse, France; Centre de Biologie Structurale (CBS), Université de Montpellier, INSERM, CNRS, 34090 Montpellier, France; Faculty of Biology, Institute of Organismic and Molecular Evolution, Johannes Gutenberg University Mainz, 55128 Mainz, Germany

## Abstract

**Motivation:**

Poly-alanine (polyA) regions are protein stretches mostly composed of alanines. Despite their abundance in eukaryotic proteomes and their association to nine inherited human diseases, the structural and functional roles exerted by polyA stretches remain poorly understood. In this work we study how the amino acid context in which polyA regions are settled in proteins influences their structure and function.

**Results:**

We identified glycine and proline as the most abundant amino acids within polyA and in the flanking regions of polyA tracts, in human proteins as well as in 17 additional eukaryotic species. Our analyses indicate that the non-structuring nature of these two amino acids influences the α-helical conformations predicted for polyA, suggesting a relevant role in reducing the inherent aggregation propensity of long polyA. Then, we show how polyA position in protein N-termini relates with their function as transit peptides. PolyA placed just after the initial methionine is often predicted as part of mitochondrial transit peptides, whereas when placed in downstream positions, polyA are part of signal peptides. A few examples from known structures suggest that short polyA can emerge by alanine substitutions in α-helices; but evolution by insertion is observed for longer polyA. Our results showcase the importance of studying the sequence context of homorepeats as a mechanism to shape their structure–function relationships.

**Availability and implementation:**

The datasets used and/or analyzed during the current study are available from the corresponding author onreasonable request.

**Supplementary information:**

Supplementary data are available at *Bioinformatics* online.

## 1 Introduction

Protein fragments with compositionally biased sequences, the so-called low complexity regions (LCRs), are present in all kingdoms of life where they perform crucial functions (Golding, 1999; Luo *et al.*, 2012; Wootton and Federhen, 1996). There are several families of LCRs depending on the sequence similarity among repeat units, the distance between adjacent repeats, and the complexity of the sequence pattern (Mier *et al.*, 2020a). Homorepeats (or polyX regions), stretches of a single amino acid, represent a very particular and eye-catching family of LCRs (Chavali *et al.*, 2020; Jorda and Kajava, 2010). Bioinformatic analyses indicate that there are homorepeats for the twenty naturally occurring amino acids, although they are not evenly distributed among the different kingdoms of life (Chavali *et al.*, 2017). While eukaryotic genomes code for ∼15% of proteins hosting at least one homorepeat, they represent less than 1% in bacteria and archaea. Local compositional biases for amino acids with a given physicochemical property in protein sequences induce different cellular localizations and enable homorepeat containing proteins to perform very specialized functions by mediating interactions with other molecules (Chavali *et al.*, 2020).

Accumulation of identical physicochemical properties can also have detrimental consequences and trigger protein aggregation and disease (Lobanov *et al.*, 2016). Concretely, eighteen severe pathologies have been described to originate from abnormal expansions of glutamine (polyQ) and alanine [poly-alanine (polyA)] tracts (nine for each family) (Darling and Uversky, 2017; Orr and Zoghbi, 2007). The toxic mechanisms and the aggregation pathways of polyQ-related diseases have been thoroughly studied both *in vivo* and *in vitro* (Saudou and Humbert, 2016; Wetzel, 2012). In parallel, proteome- and genome-wide bioinformatic analyses have revealed functional, structural and evolutionary features common to polyQ-rich proteins (Mier and Andrade-Navarro, 2018; Ramazzotti *et al.*, 2012; Totzeck *et al.*, 2017; Urbanek *et al.*, 2020a). Especially significant has been the discovery of strong and asymmetric compositional bias in polyQ flanking regions (Mier *et al.*, 2020a; Ramazzotti *et al.*, 2012), which has been experimentally associated to the propagation of secondary structure toward the homorepeat and the modulation of their aggregation propensity and toxicity (Bhattacharyya *et al.*, 2006; Escobedo *et al.*, 2019; Urbanek *et al.*, 2020a).

Conversely to polyQ, less knowledge has been accumulated on the structural, functional and pathological role of polyA stretches. PolyA is the second most abundant homorepeat in Metazoa (present in more than 2% of protein families) after polyE (Mier *et al.*, 2017); 7.8% of human proteins contain tracts with four or more consecutive alanines (Pelassa *et al.*, 2014). The pathological threshold of the specific segments triggering polyA-related diseases has been identified (Albrecht and Mundlos, 2005; Amiel *et al.*, 2004; Shoubridge and Gecz, 2012). Interestingly, it has been observed that this threshold is protein-dependent, suggesting an active role of the sequence context in modulating toxic oligomerization.

From a functional perspective, it has been shown that polyA are not mere spacers inserted in proteins, but they are engaged in protein–protein and protein–DNA interactions (Brown and Brown, 2004). Furthermore, polyA-containing proteins present frequently a nuclear localization (Albrecht and Mundlos, 2005). These observations are in line with the large percentage of these proteins (34%) annotated as transcription factors (Lavoie *et al.*, 2003). Interestingly, this percentage increases when only repeats with eight or more consecutive alanines are considered. Placed in the disordered tails of transcription factors, the functional advantages provided by polyA to this family of proteins remain to be unveiled.

Since the seminal study by Gratzer and Doty (1963), the structural properties of polyA homorepeats have attracted the biophysical community. Although this pioneering study suggested that the polyA forms a highly stable α-helix in aqueous solutions, different conclusions were subsequently derived. In a series of studies using multiple biophysical techniques, Kallenbach’s group suggested that polyA peptides are disordered with some prevalence for poly-proline II conformations (Chen *et al.*, 2004, 2007; Shi *et al.*, 2002). Importantly, these investigations were performed on short blocked model peptides, which could display different structural properties than polyA tracts in their protein context. Indeed, two recent nuclear magnetic resonance (NMR) studies have unambiguously identified three stretches with five, six and eight consecutive alanines as partially formed α-helical structures (Chen and Huang, 2020; Hong *et al.*, 2019). These contradictory observations suggest a relevant structural role of polyA flanking regions. Up to now, the amino acid enrichment in polyA flanking regions has not been systematically studied, although an enhanced occurrence of proline, glycine and serine has been observed for some of the polyA tracts of the transcription factor HOXA13 in mammals (Mortlock *et al.*, 2000). In addition to the putative role in modulating polyA secondary structure, the physicochemical nature of the flanking regions and the amino acid insertions can exert a strong influence to the protein functional and disease-related properties. For instance, neighboring residues could modulate the oligomerization propensity of polyA, which seems to proceed via the formation of α-helical clusters and/or coiled coil interactions (Pelassa *et al.*, 2014; Polling *et al.*, 2014, 2015). Difficulties in elucidating experimentally the structural propensity of homorepeats have hampered the definition of the structure–function relationship in polyA (Katti *et al.*, 2000; Urbanek *et al.*, 2020b; van der Lee *et al.*, 2014), and have promoted the application of bioinformatic approaches (Lavoie *et al.*, 2003; Pelassa *et al.*, 2014).

In the present study, we have analyzed the human proteome and 17 additional eukaryotic proteomes to study the sequence trends and structural properties in polyA and their flanking regions. These analyses provide indications on the role of polyA in mitochondrial localization as a late evolutionary trend. Moreover, our results indicate that amino acid prevalence in alanine-rich sequences regulate the structural properties of these regions, suggesting a mechanism to tune the interaction with their biological partners and the modulation of the aggregation propensity in pathologically expanded polyA tracts.

## 2 Materials and methods

### 2.1 Data retrieval and processing

We obtained the complete reference human proteome (hsa) from UniProtKB v2021_02 (UniProt Consortium, 2021), consisting of 20 614 proteins. Additionally, we downloaded the proteome of 17 other eukaryotic species from the same source (Supplementary File S1): *Mus musculus* (mmu), *Bos taurus* (bta), *Ornithorhynchus anatinus* (oan), *Taeniopygia guttata* (tgu), *Anolis carolinensis* (aca), *Xenopus tropicalis* (xtr), *Danio rerio* (dre), *Takifugu rubripes* (tru), *Branchiostoma floridae* (bfl), *Strongylocentrotus purpuratus* (spu), *Drosophila melanogaster* (dme), *Anopheles gambiae* (aga), *Apis mellifera* (ame), *Daphnia pulex* (dpu), *Caenorhabditis elegans* (cel), *Arabidopsis thaliana* (ath) and *Volvox carteri f. nagariensis* (vca).

Positional annotations [mitochondrial transit peptides (mTPs) and signal peptides] were predicted using TargetP v2.0 (Almagro Armenteros *et al.*, 2019), and the subcellular location annotation was obtained from UniProtKB v2021_02.

### 2.2 Search for polyA regions

The search for polyA regions was done with an in-house script, similar to those used in previous studies for other homorepeats (Mier *et al.*, 2017, 2020b). To locate a large number of pure polyA regions we used a lax threshold of four alanine residues in a local window of four amino acids. Once a region matching the threshold was found in a protein, it was extended until a non-Ala residue was located. We named these regions consisting of consecutive alanine residues as pure polyA. To locate impure polyA regions, regions mostly composed of Ala residues, we used a threshold of four to five alanine residues in a window of 6. As with the pure regions, once an impure polyA region was located, it was extended until the threshold was not met.

### 2.3 Secondary structure prediction

The secondary structure propensities of the polyA stretches and the 12 residues flanking them were predicted using the local structural propensity predictor (LS2P) (Estaña *et al.*, 2020). Briefly, the LS2P method splits the sequence into overlapping tripeptides, with two amino acids shared between them. Then, a database of tripeptides extracted from coil regions of experimentally determined high-resolution structures is searched to assign structural preferences to each tripeptide. In addition, to take into account the sequence context of a tripeptide *i*, the structural preferences of the two preceding (*i−2*, *i−1*) and following (*i + 1*, *i + 2*) tripeptides are also considered within an analytical equation to predict structure. The final secondary structure propensity calculated in this manner is assigned to the central amino acid of each overlapping tripeptide along the sequence. LS2P considers 27 structural classes for tripeptides, as a combination of α, *β*, and γ regions of the Ramachandran’s space (details can be found in the original study; Estaña *et al.*, 2020). In this work, we considered *ααα* (all three residues of the tripeptide in a helical conformation), *βββ* (all three residues of the tripeptide in an extended conformation), and *Others*, which groups all the other classes. Note that to avoid artifacts at the termini, only the conformational preferences of the ten preceding (−1 to −10) and following (+1 to +10) residues of the polyA are reported.

## 3 Results

### 3.1 A Survey of the polyA regions in the human proteome

PolyA or A-rich regions are general terms to denote a protein sequence with a high frequency of alanine residues. To characterize these regions, we must first accurately define them. A polyA region can be pure, if it is only composed of alanines, or impure, if it contains one or a few non-alanine residues. Following previous work, we require a minimum of four consecutive alanines for a pure polyA; for an impure polyA, we require at least four alanine residues in a six residue region (Mier *et al.*, 2017, 2020a). Longer regions with lower but significantly high frequencies of alanine also belong to the category of A-rich sequences, for example defined as compositionally biased regions (Promponas *et al.*, 2000), but are not considered in this study.

By applying these thresholds, we located 2030 pure and 6295 impure polyA regions in the human proteome (Table 1). Note that we report more polyA than Lavoie *et al.* in 2003, which used a stricter threshold (five consecutive alanine residues, 604 regions), but a similar number than Pelassa *et al.* (2014). We found pure and impure polyA regions in 1582 (7.67%) and 4398 (21.33%) human proteins, respectively. Approximately 30% of pure and 40% of impure polyA-containing proteins have more than one polyA tract. A large variability in the number of polyA-containing proteins was found when performing equivalent analyses in other 17 eukaryotic proteomes (Supplementary File S1). The percentage of proteins containing pure polyA tracts ranged from 3% (*C. elegans*) to 39% (*V. carteri*), and increased from 11% (*C. elegans* and *X. tropicalis*) to 57% (*V. carteri*) for impure homorepeats. Interestingly, no correlation was found between the number of polyA along evolution.

**Table 1.** Frequency of polyA regions in the human proteome

Pure polyA			Impure polyA		
Length	PolyA	Proteins	Length	PolyA	Proteins
4	1185	1056	5	1427	1292
>4	845	687	6	2919	2457
			>6	1949	1580
Total	2030	1582	Total	6295	4398

**Fig. 1.**
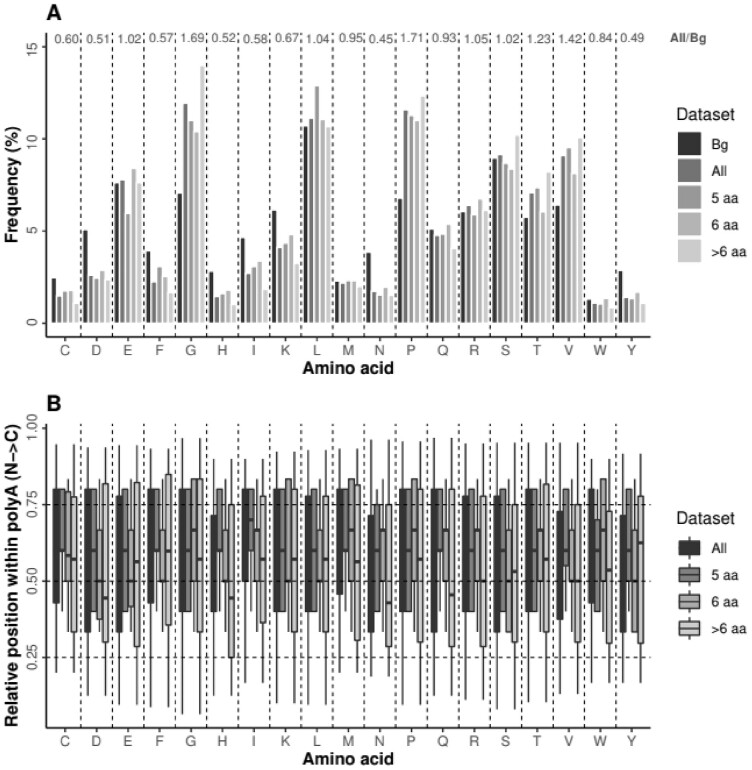
Characterization of non-Alanine residues in human impure polyA regions. (**A**) Frequency and (**B**) relative position within the impure polyA regions of non-Alanine residues in short (length = 5 aa), medium (6 aa) and long (>6 aa) polyA. To calculate the background frequency per amino acid (‘Bg’) we used the complete human proteome. The ratio All/Bg is shown on the top of panel (A) for all amino acids. The boxplots indicate second to third quartile and median

An analysis of the amino acids found as impurities in polyA regions revealed that the frequency of such residues varies with respect to their frequencies in the human proteome (Fig. 1A). On the one hand, D, H, I, N and Y are strongly depleted in polyA regions (ratio all/bg ≃ 0.50). On the other hand, G and P are enriched as impurities in polyA stretches (ratio >1.5). An evolutionary study of these trends in the 18 eukaryotic species shows that the trend of high G and P in human is not general for all species, but it is mainly shared within Amniota, equivalently to the depletion in N and I (Supplementary Fig. S1). Dipterans (*Anopheles gambiae*, *Drosophila melanogaster*) have also high G and P levels, but also higher levels of T and V, and lower R than Amniota. Depletion of C, D and aromatic residues (F, H, W, Y) can be observed across all species. This overview suggests that while some general rules may exist for the insertion of amino acids in polyA, there is appreciable inter-species variability with some trends clustered in wide taxa, which suggest some functional association. The very high levels of impurities of P and T stand out in *C. elegans* and *V. carteri*, respectively. Properties related to the specific lifestyle of these species could play a role in these cases.

Our analysis shows length-dependency in the frequency of the non-alanine residues within the polyA regions for G and P, which are more prevalent in long impure polyA regions, and for L, which is more prevalent in short polyA (Fig. 1A). Regarding the position of the impurities, we observed a tendency for them to be located in the second half of the polyA (relative position >0.5; Fig. 1B), with I in the five amino-acid (aa) long polyA as the most C-terminally positioned. Exceptions were observed in long polyA for D, H, N and Q (relative position <0.5).

### 3.2 The sequence context of polyA regions

**Fig. 2.**
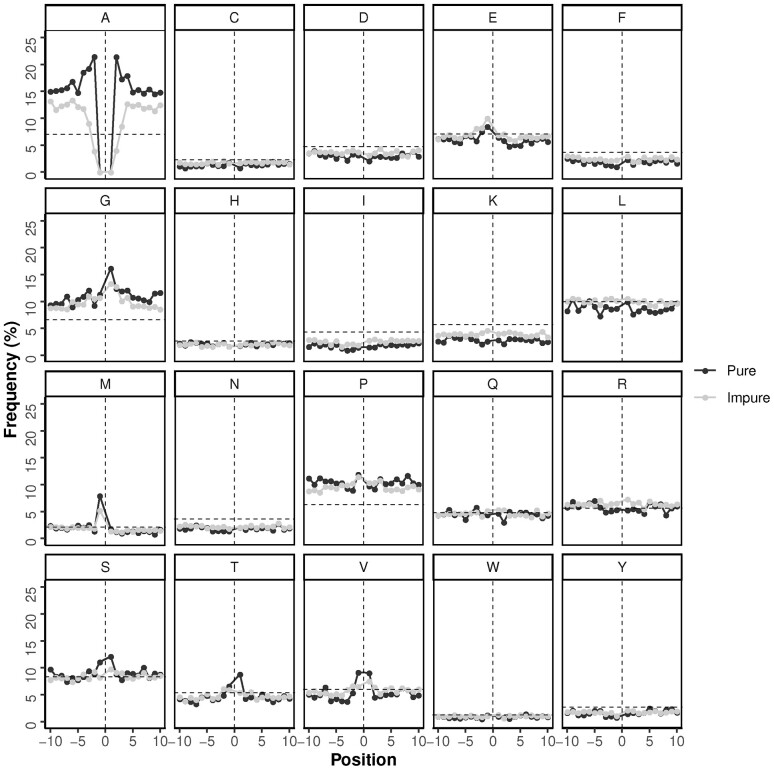
Amino acid context of polyA regions. Amino acid abundance per position of the N-terminal (−10 to −1) and C-terminal (+1 to +10) flanking regions for all pure and impure human polyA regions. Horizontal dashed lines indicate the background frequency of the corresponding amino acid in the human proteome. Vertical dashed line (position 0) indicates the position of the polyA region

The sequence context in which polyQ regions are placed is important from a structural and functional perspective, and has been extensively studied (Bhattacharyya *et al.*, 2006; Escobedo *et al.*, 2019; Ramazzotti *et al.*, 2012; Shen *et al.*, 2016; Urbanek *et al.*, 2020a). Here, we similarly investigated the context of polyA sequences, studying 10 amino acids before (−1 to −10) and after (+1 to +10) the polyA (Fig. 2). Interestingly, the regions surrounding polyA stretches are enriched in alanine residues, notably around pure tracts. This suggests that polyA tend to be contained within longer alanine-rich regions.

Most of the other amino acids display a flat distribution with values near the background frequency. There are, however, a few exceptions: I and K stand out by their low frequencies, and P and G by their high frequency. A few amino acids present higher frequencies at specific positions near the polyA, most of which are only present or more pronounced for pure polyA: G and T at position +1, M at position −1, and S and V at positions −1 and +1. E at position −1 seems to be more pronounced for impure polyA (Fig. 2). Interestingly, the general enrichment of G and P encompasses the two flanking regions and is even greater for long and pure polyA (Supplementary Fig. S2).

**Fig. 3.**
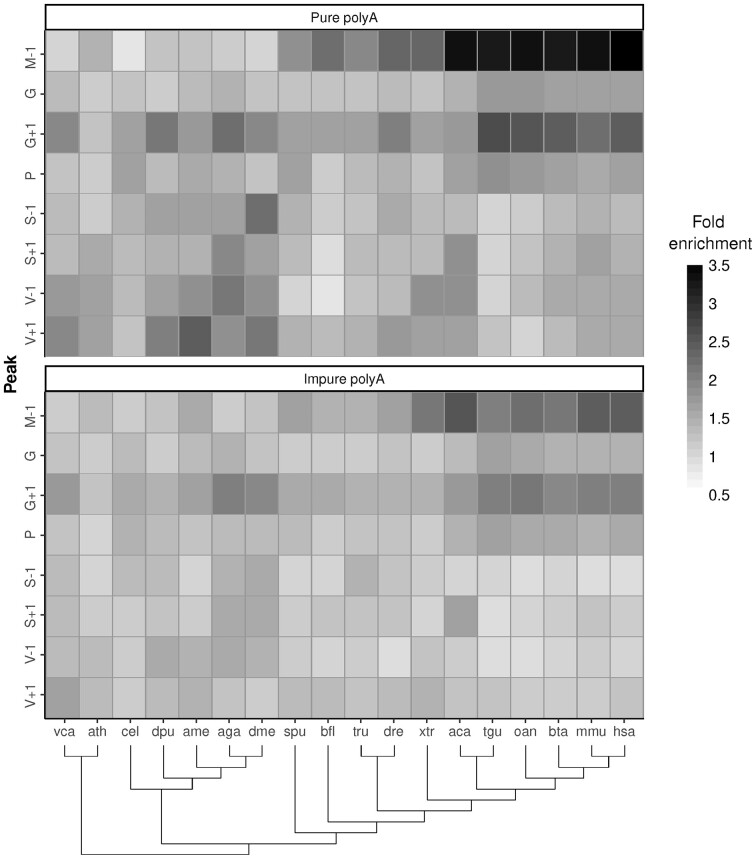
Amino acid enrichment around the polyA regions for 18 eukaryotic species. The enrichment is calculated as the frequency of an amino acid in a position compared to its frequency in the corresponding proteome. Selected positions are the ones detected as important in human proteins: glycine in position +1 (G + 1), methionine in position −1 (M−1), serine in positions −1 (S−1) and +1 (S + 1), valine in positions −1 (V−1) and +1 (V + 1), and proline and glycine in positions from −10 to +10 (P and G). For the complete species names, see Supplementary File S1. The phylogenetic tree indicates the phylogenetic relationships between species

In order to evaluate the evolutionary conservation of these trends, we performed the same type of analysis for the additional 17 additional eukaryotic species. This analysis shows that some trends found in the human proteome are conserved in all the eukaryotes analyzed, although the enrichments found were not homogeneous in all proteomes (Fig. 3). The results indicate that the peak in G + 1 is common to most species. Conversely, the M−1 peak for pure polyA is observed in Deuterostomia (from spu to hsa; Fig. 3) with higher values in Amniota (from aca to hsa; Fig. 3). Given that M and A translate from distinct codons (AUG and GCx, respectively), sequencing or translation errors could not be at the origin of this enrichment. In the next section, we discuss this case in more detail.

When analyzing the overall enrichment of P and G encompassing both flanking regions, we observed that this feature is especially significant only for Amniota. Interestingly, the enrichment in valines in the immediate residues of polyA, V−1 and V + 1, is significant for the eukaryotes non deuterostomes, from vca to dme, with the exception of cel (Fig. 3). Although less intense, similar results were observed for the enrichment of serines, S + 1 and S−1. When comparing the compositional bias in pure and impure polyA, we observed that the evolutionary trends described above are common for both sets, although the enrichment is systematically lower for impure sequences.

### 3.3 The protein context of polyA regions and their association to TPs and cellular localization

**Fig. 4.**
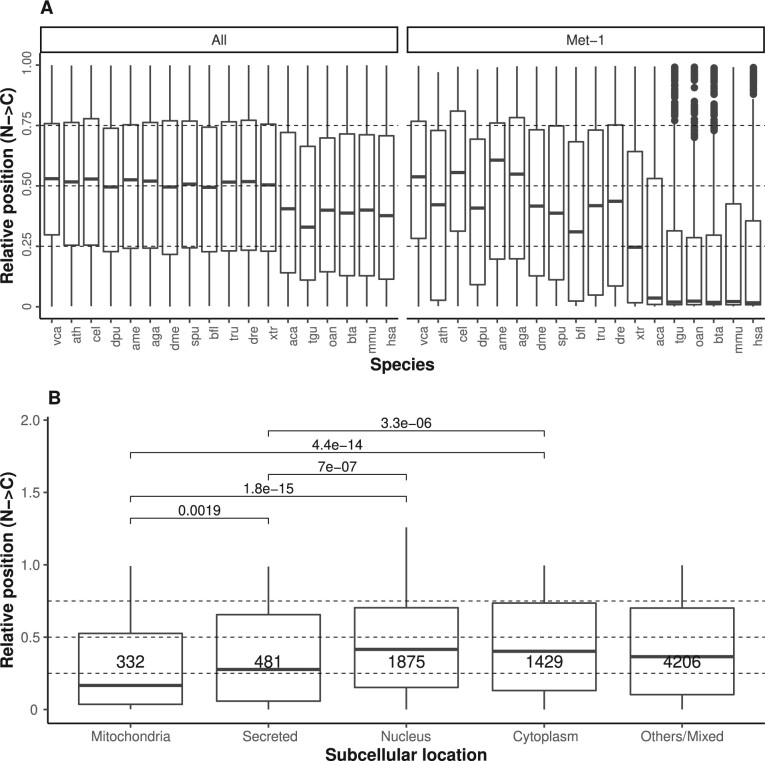
Relative position of polyA in proteins and subcellular location. (**A**) Position of polyA regions within proteins of different eukaryotic species, for all polyA (left) and for those with a methionine residue in position −1 (right). (**B**) Position of human polyA regions depending on the subcellular location of the hosting protein; subgroup ‘Other/Mixed’ includes proteins with more than one subcellular location or different from the other subgroups. PolyA from mitochondrial and secreted proteins are significantly closer to the protein N-terminal than polyA from proteins located in the nucleus (*P*-value 1.8e−15 and 7e−07, respectively) or in the cytoplasm (*P*-value 4.4e−14 and 3.3e−06, respectively; Man–Whitney U Test)

When analyzing the position of the polyA tracts in proteins, we observed that these regions have a strong bias toward the N-termini of proteins in Amniota (aca to hsa; Fig. 4A—left). This evolutionary distribution resembles the one found for methionine preceding polyA (M−1). When analyzing proteins of the M−1 group, we observed that polyA tracts are highly enriched at the N-termini of these proteins in Amniota, while no special localization was found for the other species (Fig. 4A—right). Indeed, almost two thirds of these polyA are at position 2, right after the initial methionine. These N-terminal polyA are not the sole responsible for the N-terminal bias in Amniota, since this positional bias (relative position ∼0.30–0.40) remains even when considering only polyA starting at position >2 (data not shown).

The positional bias of polyA regions in Amniota proteins suggested a putative role of these homorepeats as TPs or signal peptides (SPs), which are coding sequences labeling proteins for secretion or cellular localization (Owji *et al.*, 2018). These sequences, which are normally 25–30 residue long, often present an α-helical hydrophobic core that is the main responsible of their selective localization (Hatsuzawa *et al.*, 1997; Von Heijne, 1990). Using the TargetP v2.0 server (Almagro Armenteros *et al.*, 2019), we analyzed whether proteins with an N-terminal polyA were predicted to having TPs or SPs. To simplify the analyses for the cases in which a protein has more than one polyA region, we only took into consideration the most N-terminal one.

PolyA-containing proteins are 2-fold enriched in mTPs (Table 2), compared to proteins without any polyA. This enrichment is 5.5-fold when the polyA starts at position 2. We compared these values with those for predicted SPs, in which an enrichment is only seen in proteins with the polyA starting in positions 3–20 (1.4-fold enrichment). These observations suggest a functional role of N-terminal polyA regions in protein localization. To test this hypothesis, the subcellular localization of human polyA-containing proteins was analyzed as a function of the position of the homorepeat. In fact, mitochondrial and secreted polyA-containing proteins have their polyA regions significantly more N-terminally than nuclear and cytoplasmic proteins (Fig. 4B). Interestingly, results by subcellular location also show that polyA in mitochondrial proteins are located more N-terminally than in secreted ones. Conversely, there is no difference in the relative position of the polyA for nuclear and cytoplasmic proteins, both in the ∼0.30–0.40 range described before (Fig. 4A).

**Table 2.** Mitochondrial transit peptides (mTP) and signal peptides (SP) predicted for human proteins, considering the most N-terminal polyA region per protein

PolyA start position	Proteins	mTP	SP	%mTP	%SP
No polyA	15 784	337	2894	2.14	18.34
Any	4830	213	749	4.41	15.51
2	229	27	26	11.79	11.35
3–20	622	45	156	7.23	25.08
>20	3979	110	539	2.76	13.55

### 3.4 The sequence context of polyA regions modulates their inherent helical propensity

The compositional biases found as polyA impurities and in their flanking regions necessarily exert strong influence on the structural properties of the homorepeat. To better understand this influence, we predicted the structural propensities of the polyA fragments including the ten preceding and following residues. The structural propensities were calculated with a recently developed algorithm that enumerates the structures of overlapping three-residue fragments (tripeptides) found in a database of experimentally determined high-resolution protein structures (Estaña *et al.*, 2020). The method considers 27 structural classes and quantifies the propensity for each overlapping tripeptide segment along the sequence to be observed in each class. To simplify the interpretation of our analysis, we reduced the 27 classes into three: α-helix (*ααα*), extended (*βββ*) and *Others*, which encompasses all the other 25 conformational classes.

**Fig. 5.**
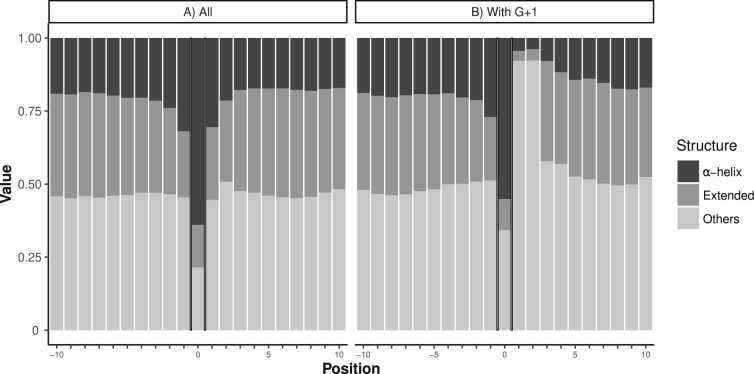
Secondary structure propensity prediction within and in the flanking regions of pure polyA. Fraction of predicted α-helical, β-sheet (extended) and Others secondary structure conformations in the N- (positions −10 to −1) and C- (positions +1 to +10) terminal flanking regions for (**A**) all pure human polyA stretches, and (**B**) only those with a glycine residue in position +1 (G + 1). Values at position 0 refer to the mean value for the polyA regions. All calculations were performed with the LS2P program

According to our approach, pure polyA regions have a strong tendency to adopt α-helical conformations (in line with NMR studies of polyA tracts in proteins; Chen and Huang, 2020; Hong *et al.*, 2019), which extend to the immediate flanking residues (Fig. 5A). Very similar results were obtained when analyzing impure polyA sequences, indicating that the thresholds used to select polyA were appropriate.

The general enrichment of G and P, two amino acids normally classified as non-structuring, in regions surrounding polyA (Fig. 2), which gets stronger for longer polyA (Supplementary Fig. S2), clearly reduces the predicted α-helical propensity of the sequence surrounding polyA. This behavior is exacerbated when the polyA regions have a G in position +1 (Fig. 5B). In this case, the glycine and the following residue adopt higher percentages of *Others* conformations than the rest of the neighboring residues of the flanking region.

Our results show that polyA and their flanking regions display opposite structural preferences and suggest that the specific sequences found around polyA could serve to restrain or limit the extent of the inherent helical structure of this repeat. The observation that longer polyA, which might be expected to form more stable helical structures, present a higher frequency of structure-breaking residues G and P is consistent with this view (Supplementary Fig. S2).

### 3.5 Structural and evolutionary information suggest various mechanisms of polyA emergence

The types of evolutionary emergence of a sequence feature can be used to obtain information about the constrains that surround its function and structure; this approach has been used to study polyQ, distinguishing cases where it is inserted or generated by glutamine substitutions (Mier and Andrade-Navarro, 2020). To identify mechanisms by which polyA emerges in evolution in the context of protein structures, we searched the Protein Data Bank (Burley *et al.*, 2021) to investigate particular examples of experimentally solved structures of polyA regions in human proteins. Additionally, we used the online tool dAPE (Mier and Andrade-Navarro, 2017), which displays precomputed results comparing the position and type of homorepeats in protein families, to verify the evolutionary conservation of polyA in very distant species. While there are many structures of human proteins containing polyA, these regions tend to be absent from the modeled parts, suggesting their flexible nature, particularly for longer ones (six or more alanines).

Here, we present some examples of these long unmodeled polyA. KDM1A (UniProtKB: O60341) has a pure polyA of length 12 starting at position 7, conserved down to *B. taurus* but not in *Gallus gallus* and beyond. COPS6 (UniProtKB: Q7L5N1) has a pure polyA of length 9 conserved down to *X. tropicalis* but not in *T. rubripes* and beyond. SNRPB (UniProtKB: P14678) has a region A_8_TA at position 151 conserved down to *T. rubripes* but not in *Ciona intestinalis* and beyond. MAPK1 (UniProtKB: P28482) has a region A_6_GA at position 2 and is conserved in *X. laevis* but not in *T. rubripes* and beyond. These evolutionary patterns suggest that establishment in a taxonomic range is very stable and becomes fixed once it occurs. The fact that these polyA were absent from the corresponding 3D structures suggests their flexible nature. We found one partial exception for MZT1 (UniProtKB: Q08AG7), which has a polyA of length 10 at position 7 (AGA_8_), conserved down to *B. taurus*. In the available structure of this protein (PDB: 6M33; Wieczorek *et al.*, 2020), the last five alanines of the polyA are part of a modeled 24-residue long α-helix.

**Fig. 6.**
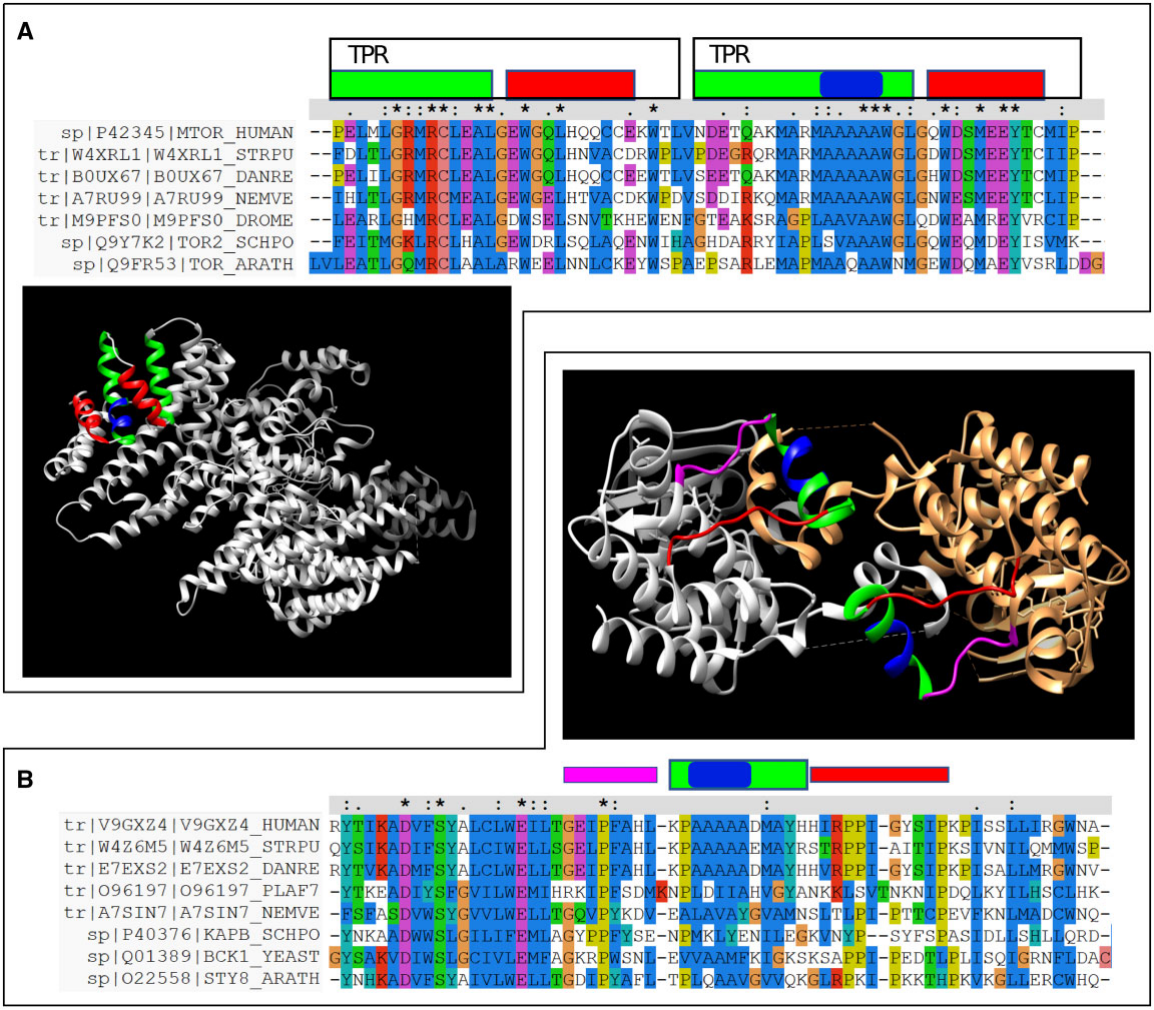
Structures of polyA regions in human proteins. (**A**) MTOR (UniProtKB: P42345) has a pure five-residue long polyA at position 1516 in the first helix of a TPR tandem repeat (PDB: 4JSV; Yang *et al.*, 2013). (**B**) TNNI3K (UniProtKB: Q59H18) has a five-residue long pure polyA at position 671, which is part of an α-helix (PDB: 6B5J; Philp *et al.*, 2018); the two subunits that form the homodimer are shown, in white and orange. Orthologs of the human proteins were obtained using ProteinPathTracker (Mier *et al.*, 2018), proteins were aligned with MUSCLE (Edgar, 2004), and alignments were displayed using ClustalW (Larkin *et al.*, 2007). The boxes above the alignments correspond to the structural elements similarly colored in the structures (A color version of this figure appears in the online version of this article)

We present two illustrative examples of pure polyA of length 5 within solved structures. To study in detail their conservation in very distant species, we obtained sets of selected orthologs using multiple evolutionary paths from the ProteinPathTracker online tool (Mier *et al.*, 2018). In both cases, the polyA is part of a larger helix. Furthermore, the alignment with very distant orthologs (including proteins from plant and fungi) suggests that both polyA did not emerge by insertion, but by successive alanine substitution (Fig. 6). This hypothesis is exemplified for the case of MTOR (UniProtKB: P42345), which displays a pure polyA of length 5 at position 1516 in the first helix of a TPR tandem repeat (PDB: 4JSV; Yang *et al.*, 2013) (Fig. 6A). Note that TPR repeats are composed of two anti-parallel helices (Das *et al.*, 1998). Tandem repeats such as TPR emerge by tandem duplication of an ancestral unit and often diverge rapidly in sequence so that their detection becomes difficult by sequence analysis even if their structures maintain the repetitive pattern (Andrade *et al.*, 2001; Kajava, 2012). The fact that a polyA occurs within a TRP unit indicates that it must have been originated by replacing consecutive residues by alanines within the α-helix.

The second example suggests a function that could be more general and exemplifies the advantages (and potential dangers) of polyA within a flexible context. In TNNI3K (UniProtKB: Q59H18; PDB: 6B5J; Philp *et al.*, 2018), a pure polyA of length 5 at position 671 is part of an α-helix, where the flanking regions are rich in prolines and adopt a coil structure, placing the helix away from the rest of the protein (Fig. 6B). In the crystallographic structure, two proteins actually intertwine forming a homodimer that is grappled by the polyA-containing helix.

These examples from solved structures confirm the α-helical nature of polyA and their increased flexibility with the length. Conservation over long evolutionary distances, even for the short polyA, suggests a relevant functional role. Exemplary structures indicate that polyA can emerge by successive residue substitutions in α-helices. Furthermore, the case of TNNI3K suggests that they could form flexible hooks with the capacity to adopt a rigid helical structure upon the interaction with a partner, resembling the coiled–coil interactions leading to the aggregation of abnormally expanded polyA (Pelassa *et al.*, 2014; Polling *et al.*, 2015).

To determine the extent to which polyA may be generated by insertions or substitutions, we selected orthologs of 24 pure polyA with 15 or more residues from 23 proteins (Supplementary File S2). Results show that polyA generation by insertion is much more frequent than polyA generation by substitution in long polyA tracts. Indeed, out of the 24 long polyA tracts, 13 were generated by an insertion mechanism, while only 2 were generated by substitution; in 9 cases, a mixture of both mechanisms was observed.

## 4 Discussion

This study shows that the sequence context in which a polyA region is located in a protein plays an important role at functional, evolutionary and structural levels. From a functional perspective, polyA had been associated to protein–protein and protein–DNA interactions (Brown and Brown, 2004), and had been assigned as important elements in transcription factors (Albrecht and Mundlos, 2005). Here, we describe an additional function of polyA regions as part of TPs and SPs in Amniota: either in mTPs when polyA regions start at position 2 of the protein, just after the initial methionine, or as part of SPs when the polyA is placed in positions 3–20. Their involvement in TPs is shared by other homorepeats, being polyL the most common one (Labaj *et al.*, 2010; Mier *et al.*, 2017). The hydrophobic character of alanine and its inherent propensity to adopt α-helical conformations are in line with the trends observed for SPs and TPs (Hatsuzawa *et al.*, 1997; Owji *et al.*, 2018).

The lack of correlation between the number of polyA along evolution (for example, the extremely high frequency found in the green alga *V. carteri*; Supplementary File S1) suggests that in addition to its function in biomolecular interactions, polyA must have other functions specific to species lifestyle and environment. Similarly, species variability with higher frequencies in unicellular eukaryotic species has been found for other compositionally biased protein features, including other homorepeats (e.g. polyN is highly abundant in *Plasmodium falciparum* and *Dictyostelium discoideum*; Mier *et al.*, 2017), and predicted intrinsically disordered regions are highly abundant in *Chlamydomonas reinhardtii* (Kastano *et al.*, 2020), a green alga evolutionarily related to *V. carteri*.

In addition to the previously described enrichment of polyA regions in position 2 in Amniota proteins, the prevalence of some amino acids in polyA flanking regions seems to be evolutionary conserved. G and P are systematically found in the proximities of polyA tracts, although they are found more often in Amniota. Interestingly, the immediate position after the polyA is the preferred location for G. These observations suggest a functional benefit of associating polyA with G and P, which are two non-structuring amino acids, that is specially exploited in Amniota. Other identified amino acid enrichments seem to be localized in specific positions with respect to the polyA region. S and V in the immediate positions on both sides of the polyA are especially abundant in metazoans. We hypothesize that the amino acid enrichments identified here are related with the structural influence that they exert to polyA regions (see below). The heterogeneous evolutionary distribution of these amino acid enrichments suggests that the functional role of polyA regions has been modified or enlarged in multiple independent evolutionary events, each of them influencing the composition of the flanking regions.

The sequence context of polyA regions has a strong influence on their structural properties. According to our predictions, polyA regions in the context of proteins tend to adopt α-helical conformations, in line with several biophysical studies (Chen and Huang, 2020; Gratzer and Doty, 1963; Hong *et al.*, 2019). While this conformation is needed for their function as interactor hubs, it may lead to aggregation if polyA regions are expanded beyond a certain threshold (Bernacki and Murphy, 2011), as described for nine developmental and neurodegenerative diseases (Albrecht and Mundlos, 2005; Amiel *et al.*, 2004; Shoubridge and Gecz, 2012). Indeed, the aggregation mechanism of polyA is triggered by the formation of helical contacts, most probably through coiled-coil structures (Pelassa *et al.*, 2014; Polling *et al.*, 2015). In this context, the presence of α-helix-breaking residues would modulate the length and stability of polyA helical conformations, exerting a protective role to aggregation. In line with this hypothesis, the enrichment in P and G is polyA length-dependent and, although this mechanism is shared by all eukaryotes, it is especially relevant for amniota. The finding that also P and G are the two most frequent amino acids within long impure polyA (Fig. 1A) is consistent with this hypothesis. Their higher frequency in the second half of the polyA (Fig. 1B) suggests a directional preference in their function as helix breakers and agrees with the high frequency of G at the C-terminus of polyA, enhanced in pure polyA tracts (G + 1; Fig. 3).

Our hypothesis on the protecting role of helix-breaking amino acids in polyA is also substantiated by clinical and biochemical studies. In a clinical study of two brothers with an unusual gene duplication in ARX, both individuals presented a tract with 23 alanines interrupted by a glycine, differing from the most common expanded ARX version, which contains 21 alanines (Demos *et al.*, 2009). Interestingly, these individuals, despite having a larger number of alanines in the homorepeat, presented a milder phenotype than the most common expansion of the gene, suggesting a relevant role for the intercalated glycine in the pathogenicity of ARX.

In a recent study, it was shown that the perturbation of the helical stability of the polyQ/polyA repeat in RUNX2 by substituting either certain glutamines and alanines by other amino acids had a direct impact on the structure, aggregation propensity, localization and toxicity of the protein (Pelassa *et al.*, 2014). When leucine and valine were introduced in the repeat, the α-helical content of the protein increased, concomitantly enhancing its aggregation propensity and localizing the protein in the cytosol. Conversely, the introduction of the structure-breaking proline reduced the helical content of the RUNX2 as well as its transcriptional activity. In the context of our study, the specific amino acid enrichments found as impurities or in the flanking regions induce opposite effects. While the enrichment of valine as impurity, and valine and serine in the immediacy of polyA regions, will have a stabilizing effect for helical conformations, the presence of glycine and/or proline will reduce or destroy the polyA intrinsic structural propensity. Interestingly, more structurally neutral amino acids have been depleted from polyA-rich regions. Thus, the length and stability of the helical polyA tracts tune the function and aggregation propensity of their hosting proteins.

Structural and conservation information suggests that while all polyA can be conserved across very long evolutionary distances, longer polyA are more flexible (and more absent from modeled regions in solved 3D structures) than shorter polyA. Few existing structures from the PDB identified in this study confirm the α-helical propensity and conservation mechanisms of polyA tracts. Furthermore, the analysis of their orthologs suggests that polyA can emerge by hijacking existing α-helical content by successive alanine substitution of consecutive residues. Given the polyA structural propensity, providing the polyA with flexible flanking regions could facilitate the function of this homorepeat in protein interactions, where the polyA could further rigidify upon partner recognition. However, the insertion mechanism, involving the rapid appearance of several consecutive alanines, seems to be preferred in long and evolutionarily stable polyA tracts. The sudden emergence of these fragments suggests the incorporation of novel activities to the protein or an enhanced performance in its function.

The structural control exerted to polyA flanking regions seems stronger than the one described for polyQ regions, probably due to the enhanced α-helical propensity of alanine with respect to glutamine. For polyQ tracts, it has been shown that the number of proline residues after the polyQ region is correlated with the polyQ length and that it also exerts a protective role (Bhattacharyya *et al.*, 2006; Urbanek *et al.*, 2020a). However, no such α-helix-breaking behavior is found in the flanking region preceding the polyQ. This difference is probably related to the structural features of both homorepeats. While polyQ requires a coupling with the upstream flanking region to adopt a stable helical structure (Escobedo *et al.*, 2019; Urbanek *et al.*, 2020a), polyA seems to be inherently structured. Consequently, the mechanisms put in place to minimize aggregation and disease between both homorepeats differ. In line with these observations, the pathogenic threshold of polyA regions, although different for each disease-associated protein, is systematically smaller than for polyQ, 12–27 and 21–55 for polyA and polyQ, respectively (Darling and Uversky, 2017), which could also explain the need for a stronger flanking region structural control.

One interesting aspect that we did not approach in this work is the study of the genomic sequences corresponding to polyA regions, specifically to assess whether the enrichment of certain types of amino acids within or in the vicinity of polyA corresponds to codons that are one mutational step away from alanine codons. Such a study should provide insights into the genetic mechanisms by which polyA arise in a controlled sequence environment in proteins.

Our findings suggest a capital role of sequence context in defining the structural features of homorepeats that, in turn, modulates their function while controlling their aggregation propensity. Structural mechanisms exerted by flanking regions are expected to apply not only to disease-related homorepeats, but also to other homorepeats through interactions that will be amino acid dependent and that remain to be explored.

## Funding

This work has been supported by the European Union’s Horizon 2020 under grant agreements No: 778247, No: 823886 and No: 648030; Labex EpiGenMed ANR-10-LABX-12-01; and MUSE-App 2021 Ondine ANR-16-IDEX-0006 awarded to PB. JC was partially supported by the French National Research Agency through grant number ANR-19-P3IA-0004.

*Conflict of Interest*: none declared.

## Supplementary Material

btac610_Supplementary_Data
