# Supplementary material for: The sequence context in poly-alanine regions: structure, function and conservation
Source: Bioinformatics. 2022 Sep 15;38(21):4851–8. doi: 10.1093/bioinformatics/btac610 (PMC9620824; doi:10.1093/bioinformatics/btac610)
Supplement: btac610_Supplementary_Data [file btac610_supplementary_data.zip › SupplFile2.pdf]

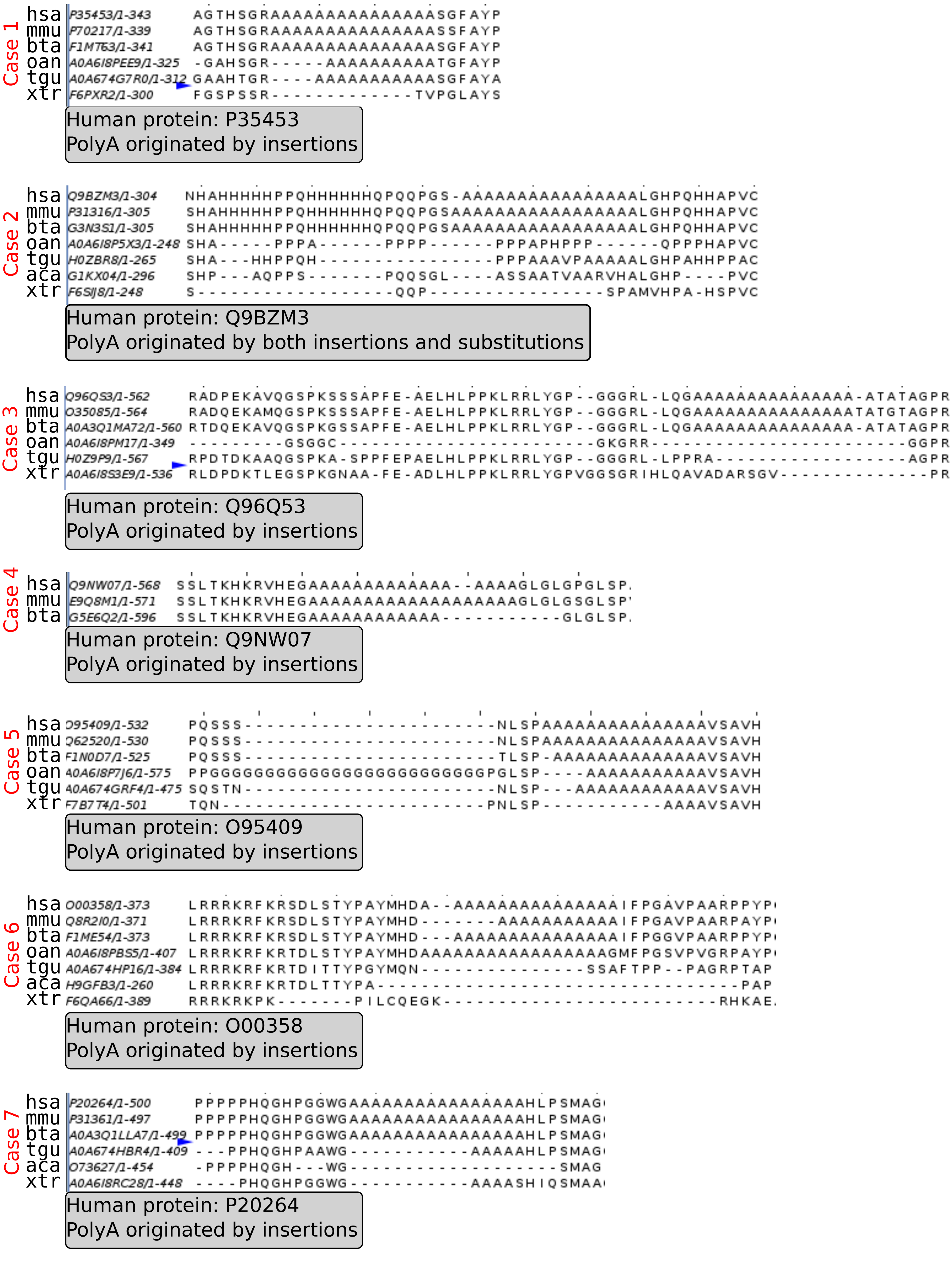

Case 1
hsa P35453/1-343 AGTHSGRAAAAAAAAAAAAAAAASGFAYP
mmu P70217/1-339 AGTHSGRAAAAAAAAAAAAAAAASSFAYP
bta F1MT63/1-341 AGTHSGRAAAAAAAAAAAAAAAASGFAYP
oan A0A6I8PEE9/1-325 -GAHSGR-----AAAAAAAAAATGFAYP
tgu A0A674G7R0/1-312 GAAHTGR----AAAAAAAAAAAASGFAYA
xtr F6PXR2/1-300 FGSPSSR-------------TVPGLAYS
Human protein: P35453
PolyA originated by insertions
Case 2
hsa Q9BZM3/1-304 NHAHHHHHPPQHHHHHHQPQQPGS-AAAAAAAAAAAAAAAALGHPQHHAPVC
mmu P31316/1-305 SHAHHHHHPPQHHHHHHQPQQPGSAAAAAAAAAAAAAAAAALGHPQHHAPVC
bta G3N3S1/1-305 SHAHHHHHPPQHHHHHHQPQQPGSAAAAAAAAAAAAAAAAALGHPQHHAPVC
oan A0A6I8P5X3/1-248 SHA-----PPPA------PPPP------PPPAPHPPP------QPPPHAPVC
tgu H0ZBR8/1-265 SHA---HHPPQH----------------PPPAAAVPAAAAALGHPAHHPPAC
aca G1KX04/1-296 SHP---AQPPS-------PQQSGL----ASSAATVAARVHALGHP----PVC
xtr F6SJJ8/1-248 S------------------QQP-----------------SPAMVHPA-HSPVC
Human protein: Q9BZM3
PolyA originated by both insertions and substitutions
Case 3
hsa Q96QS3/1-562 RADPEKAVQGSPKSSSAPFE-AELHLPPKLRRLYGP--GGGRL-LQGAAAAAAAAAAAAAAA-ATATAGPR
mmu O35085/1-564 RADQEKAMQGSPKSSSAPFE-AELHLPPKLRRLYGP--GGGRL-LQGAAAAAAAAAAAAAAAATATGTAGPR
bta A0A3Q1MA72/1-560 RTDQEKAVQGSPKGSSAPFE-AELHLPPKLRRLYGP--GGGRL-LQGAAAAAAAAAAAAAAA-ATATAGPR
oan A0A6I8PM17/1-349 ---------GSGGC-----------------------GKGRR-----------------------GGPR
tgu H0Z9P9/1-567 RPDTDKAAQGSPKA-SPPFEPAELHLPPKLRRLYGP--GGGRL-LPPRA-----------------AGPR
xtr A0A6I8S3E9/1-536 RLDPDKTLEGSPKGNAA-FE-ADLHLPPKLRRLYGPVGGSGRIHLQAVADARSGV-------------PR
Human protein: Q96Q53
PolyA originated by insertions
Case 4
hsa Q9NW07/1-568 SSLTKHKRVHEGAAAAAAAAAAAAA--AAAAGLGLGPGLSP
mmu E9Q8M1/1-571 SSLTKHKRVHEGAAAAAAAAAAAAAAAAAAAGLGLGSGLSP
bta G5E6Q2/1-596 SSLTKHKRVHEGAAAAAAAAAAAA-----------GLGLSP
Human protein: Q9NW07
PolyA originated by insertions
Case 5
hsa O95409/1-532 PQSSS-----------------------NLSPAAAAAAAAAAAAAAAAVSAVH
mmu Q62520/1-530 PQSSS-----------------------NLSPAAAAAAAAAAAAAAAAVSAVH
bta F1N0D7/1-525 PQSSS-----------------------TLSP-AAAAAAAAAAAAAAAVSAVH
oan A0A6I8P7J6/1-575 PPGGGGGGGGGGGGGGGGGGGGGGGGPGLSP----AAAAAAAAAAAVSAVH
tgu A0A674GRF4/1-475 SQSTN-----------------------NLSP---AAAAAAAAAAAAVSAVH
xtr F7B7T4/1-501 TQN------------------------PNLSP-----------AAAAVSAVH
Human protein: O95409
PolyA originated by insertions
Case 6
hsa O00358/1-373 LRRRKRFKRSDLSTYPAYMHDA--AAAAAAAAAAAAAAAIFPGAVPAARPPYP
mmu Q8R2I0/1-371 LRRRKRFKRSDLSTYPAYMHD-------AAAAAAAAAAAIFPGAVPAARPAYP
bta F1ME54/1-373 LRRRKRFKRSDLSTYPAYMHD---AAAAAAAAAAAAAAAIFPGGVPAARPPYP
oan A0A6I8PBS5/1-407 LRRRKRFKRTDLSTYPAYMHDAAAAAAAAAAAAAAAAAGMFPGSVPVGRPAYP
tgu A0A674HP16/1-384 LRRRKRFKRTDITTYPGYMQN---------------SSAFTPP--PAGRPTAP
aca H9GFB3/1-260 LRRRKRFKRTDLTTYPA---------------------------------PAP
xtr F6QA66/1-389 RRRKRKPK------PILCQEGK------------------------RHKAE
Human protein: O00358
PolyA originated by insertions
Case 7
hsa P20264/1-500 PPPPPHQGHPGGWGAAAAAAAAAAAAAAAAHLPSMAG
mmu P31361/1-497 PPPPPHQGHPGGWGAAAAAAAAAAAAAAAAHLPSMAG
bta A0A3Q1LLA7/1-499 PPPPPHQGHPGGWGAAAAAAAAAAAAAAAAHLPSMAG
tgu A0A674HBR4/1-409 ---PPHQGHPAAWG-----------AAAAAHLPSMAG
aca O73627/1-454 -PPPPHQGH---WG--------------------SMAG
xtr A0A6I8RC28/1-448 ----PHQGHPGGWG-----------AAAASHIQSMAA
Human protein: P20264
PolyA originated by insertions

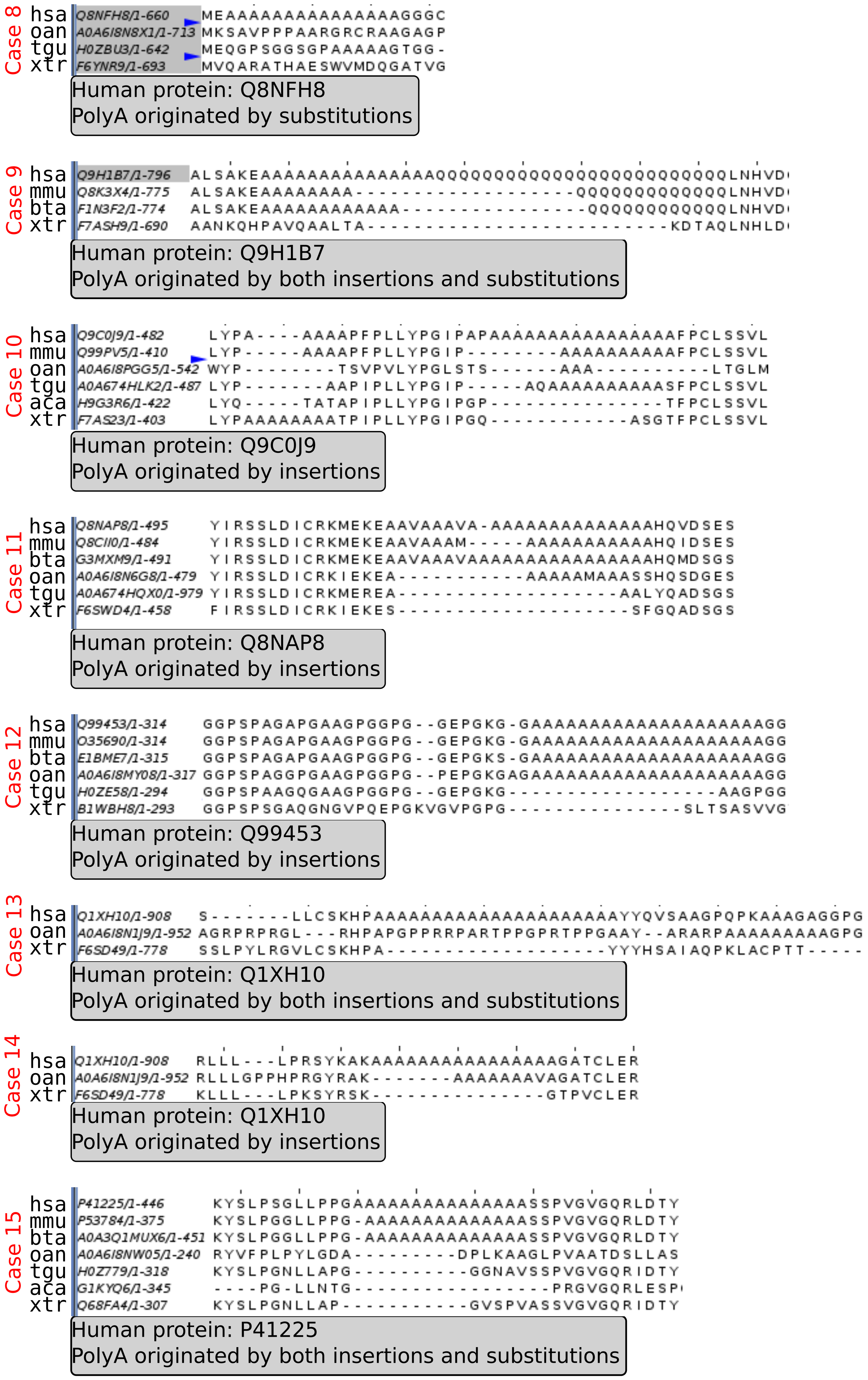
Case 8
hsa Q8NFH8/1-660 MEAAAAAAAAAAAAAAAAGGGC
oan A0A6I8N8X1/1-713 MKSAVPPPAARGRCRAAGAGP
tgu H0ZBU3/1-642 MEQGPSGGSGPAAAAAGTGG-
xtr F6YNR9/1-693 MVQARATHAESWVMDQGATVG
Human protein: Q8NFH8
PolyA originated by substitutions
Case 9
hsa Q9H1B7/1-796
mmu Q8K3X4/1-775
bta F1N3F2/1-774
xtr F7ASH9/1-690
Human protein: Q9H1B7
PolyA originated by both insertions and substitutions
Case 10
hsa Q9C0J9/1-482
mmu Q99PV5/1-410
oan A0A6I8PGG5/1-542
tgu A0A674HLK2/1-487
aca H9G3R6/1-422
xtr F7AS23/1-403
Human protein: Q9C0J9
PolyA originated by insertions
Case 11
hsa Q8NAP8/1-495
mmu Q8CII0/1-484
bta G3MXM9/1-491
oan A0A6I8N6G8/1-479
tgu A0A674HQX0/1-979
xtr F6SWD4/1-458
Human protein: Q8NAP8
PolyA originated by insertions
Case 12
hsa Q99453/1-314
mmu O35690/1-314
bta E1BME7/1-315
oan A0A6I8MY08/1-317
tgu H0ZE58/1-294
xtr B1WBH8/1-293
Human protein: Q99453
PolyA originated by insertions
Case 13
hsa Q1XH10/1-908
oan A0A6I8N1J9/1-952
xtr F6SD49/1-778
Human protein: Q1XH10
PolyA originated by both insertions and substitutions
Case 14
hsa Q1XH10/1-908
oan A0A6I8N1J9/1-952
xtr F6SD49/1-778
Human protein: Q1XH10
PolyA originated by insertions
Case 15
hsa P41225/1-446
mmu P53784/1-375
bta A0A3Q1MUX6/1-451
oan A0A6I8NW05/1-240
tgu H0Z779/1-318
aca G1KYQ6/1-345
xtr Q68FA4/1-307
Human protein: P41225
PolyA originated by both insertions and substitutions

Case 16

hsa	Q8TAU0/1-364	VGASAYSYNSFPAYGYGNSAAAAAAAAAAAAAAAAYSSSYGC
mmu	P97334/1-362	VGAGAYSYNSFPAYGYGNSAAAAAAAAAAAAAAAAYSGSYGC
bta	Q3ZC90/1-367	VGAGAYSYNSFPAYGYGNSAAAAAAAAAAAAAAAAYSGSYGC
oan	A0A6I8PJ79/1-499	VGANPYSYNAFPAYGYGNS-AAAAAAAAAAAAAAAYGGNYGC
tgu	H0ZGI5/1-327	---GPYSYNGFPAYGYGN--------------AASYNPGYGC
xtr	A0A6I8Q8L6/1-328	VTASPYSYNSYPAYSYNN--------------SPSYNTNYNC

Human protein: Q8TAU0
PolyA originated by insertions

Case 17

hsa	Q9H2Z4/1-354	MQPSHAMAGHNAAAAAAAA-AAAAAAAATYHM
mmu	Q9EQM3/1-354	MQPPHAMAGHNAAAAAAAA-AAAAAAAATYHM
bta	F1MKX5/1-347	MQPPHAMAGHN-AAAAAAA-AAAAAAAATYHM
oan	A0A6I8NEM3/1-378	MQPPHVMAGHS----AVVA-AAAAAAAASYHM
tgu	H0ZP44/1-366	MQ-QHPM-GHN----GTVT--------AAYHM
aca	G1K9J1/1-382	MQ-QHPMGGHN----GTFSVSAAAAAAAAYHM
xtr	A0A6I8QI72/1-343	MQ-QHSM-GHN----ATVT--------TTYHM

Human protein: Q9H2Z4
PolyA originated by both insertions and substitutions

Case 18

hsa	P31271/1-388	AASAYS------SAPGEAPPSAAAAAAAAAAAAAAAAAASSSGGPGPAG
mmu	Q62424/1-386	AAAAYS------SAPGEAPPSAAAAAAAAAAAAAAAAAASSSGGPGPAG
bta	E1BI23/1-388	AAAAYS------SAPGEAPPSAAAAAAAAAAAAAAAAAASSSGGPGPAG
oan	A0A6I8NA98/1-410	--AAYSGPGEPGSGSGSGSGSGSAASAPGAPGAAPGAAPGAAPGAAPGA
tgu	H0YXI5/1-305	SAAAYT-----------------SSEAPAAGMAEPA-------------

Human protein: P31271
PolyA originated by substitutions

Case 19

hsa	P0CG40/1-484	ETPRSESPDLILHDSGVSAARAAAAAAAAAAAAAAAASAGGKE
mmu	Q64HY3/1-484	ETPRSESPDLILHDSGVSAARAAAAAAAAAAAAAAAASAGGKE
bta	E1BC72/1-481	ETPRSESPDLILHDSGVSAAR---AAAAAAAAAAAAASAGGKE
oan	A0A6I8NGQ8/1-683	ETPRSESPDLILHDSGVRARP------SPAGLPGNCPGRPGRP
tgu	A0A674HSL0/1-458	ETPRSESPDLILHE-GVGAAT----------------RGPGKE
aca	H9GAB3/1-458	ETPRSESPDLLLHE------------------------ARGKD
xtr	Q0VA40/1-422	ETPRSESPELIME--GVR---------------------PGKD

Human protein: P0CG40
PolyA originated by both insertions and substitutions

Case 20

hsa	Q9HAH7/1-460	--RAGEEGP-RPTKESVRVKEERKEEAAAAAAAA-------------AAAAAAAAAAA------TGPQGLHL
mmu	Q8R089/1-466	--RAGEEGA-RPAKESVRVKEERKEEAAAAAAAA-------------AAAAAAAAAAAAAAAATTGPQGLHL
bta	A0A3S5ZPP8/1-981	--RAGEEGA-RPTKESVRVKEERKEEAAAAAAAA-------------AAAAAAAAAAA-----TTGPQGLHL
oan	A0A6I8PPF2/1-1135	PHRNGSSLP-RAALPPTRLALSLQGPLVLPTAAAGLPGCPQIAGRGGGRAASQGAGEGEGGAEGGGPRG---
xtr	A0A6I8SXA1/1-894	AARHGSSSDIKPRLTPDHSKVKVKEEPEVLTFEVGRSPVP----------------------------GLHL

Human protein: Q9HAH7
PolyA originated by both insertions and substitutions

Case 21

hsa	Q13950/1-521	QPGKMSDVSPVVAAQQQQQQQQQQQQQQQQQQQQQQQQE-------AAAAAAAAAAAAAAAA--AVPRL
mmu	Q08775/1-607	QPGKMSDVSPVVAAQQQQQQQQQQQQQQQQQQQQQQQQQQQQQQEAAAAAAAAAAAAAAAAA--AVPRL
bta	A0A3Q1MKY5/1-518	QPGKMSDVSPVVAAQQQQQQQQQQQQQQQQQQQQQQE----------AAAAAAAAAAAAAAA--AVPRL
oan	A0A6I8N4E7/1-508	SPGKMSETLPLAGQDG---GGAALLGGKL
tgu	H0Z2R6/1-472	SPGKMSEPLALPGAEH---GGA--LPGKM
aca	G1KN41/1-472	QPGKMSDVSP---AQQQQSQQQQQQHPE--------------------------------AA--AAPRL

Human protein: Q13950
PolyA originated by insertions

Case 22

hsa	O60346/1-1717	MEPAAAATVQRLPELGREDRASAPAAAAAAAAAAAAAAALAAAAGGGRS-
mmu	Q8CHE4/1-1687	MEPAAAAPAQRLADPTGEDQALA-----------------AAAAEGGRC-
bta	A0A3Q1N224/1-1538	MEPAAA-----------ETRAEG-----------------RAAAGG----
tgu	H0YUW9/1-1630	MEPAQ----QRAAE--------------------------APAAGSGRGE

Human protein: O60346
PolyA originated by insertions

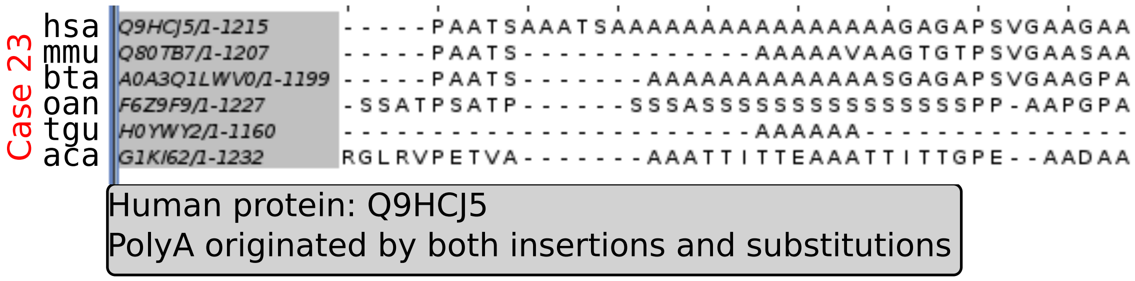
Case 23
hsa Q9HCJ5/1-1215 -----PAATSAAATSAAAAAAAAAAAAAAAAAGAGAPSVGAAGAA
mmu Q80TB7/1-1207 -----PAATS-------------AAAAAVAAGTGTPSVGAASAA
bta A0A3Q1LWV0/1-1199 -----PAATS-------AAAAAAAAAAAAASGAGAPSVGAAGPA
oan F6Z9F9/1-1227 -SSATPSATP------SSSASSSSSSSSSSSSSSSPP-AAPGPA
tgu H0YWY2/1-1160 -----------------------AAAAAA---------------
aca G1KI62/1-1232 RGLRVPETVA-------AAATTITTEAAATTITTGPE--AADAA
Human protein: Q9HCJ5
PolyA originated by both insertions and substitutions

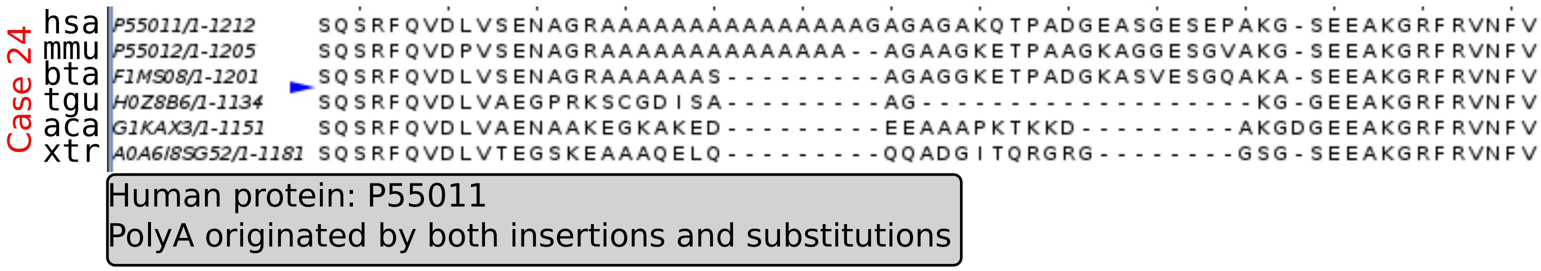
Case 24
hsa P55011/1-1212 SQSRFQVDLVSENAGRAAAAAAAAAAAAAAAAAGAGAGAKQTPADGEASGESEPAKG-SEEAKGRFRVNFV
mmu P55012/1-1205 SQSRFQVDPVSENAGRAAAAAAAAAAAAAAA--AGAAGKETPAAGKAGGESGVAKG-SEEAKGRFRVNFV
bta F1MS08/1-1201 SQSRFQVDLVSENAGRAAAAAAS---------AGAGGKETPADGKASVESGQAKA-SEEAKGRFRVNFV
tgu H0Z8B6/1-1134 SQSRFQVDLVAEGPRKSCGDISA---------AG------------------KG-GEEAKGRFRVNFV
aca G1KAX3/1-1151 SQSRFQVDLVAENAAKEGKAKED---------EEAAAPKTKKD---------AKGDGEEAKGRFRVNFV
xtr A0A6I8SG52/1-1181 SQSRFQVDLVTEGSKEAAAQELQ---------QQADGITQRGRG--------GSG-SEEAKGRFRVNFV
Human protein: P55011
PolyA originated by both insertions and substitutions
