# Supplementary figures and images for: The sequence context in poly-alanine regions: structure, function and conservation
Source: Bioinformatics. 2022 Sep 15;38(21):4851–8. doi: 10.1093/bioinformatics/btac610 (PMC9620824; doi:10.1093/bioinformatics/btac610)

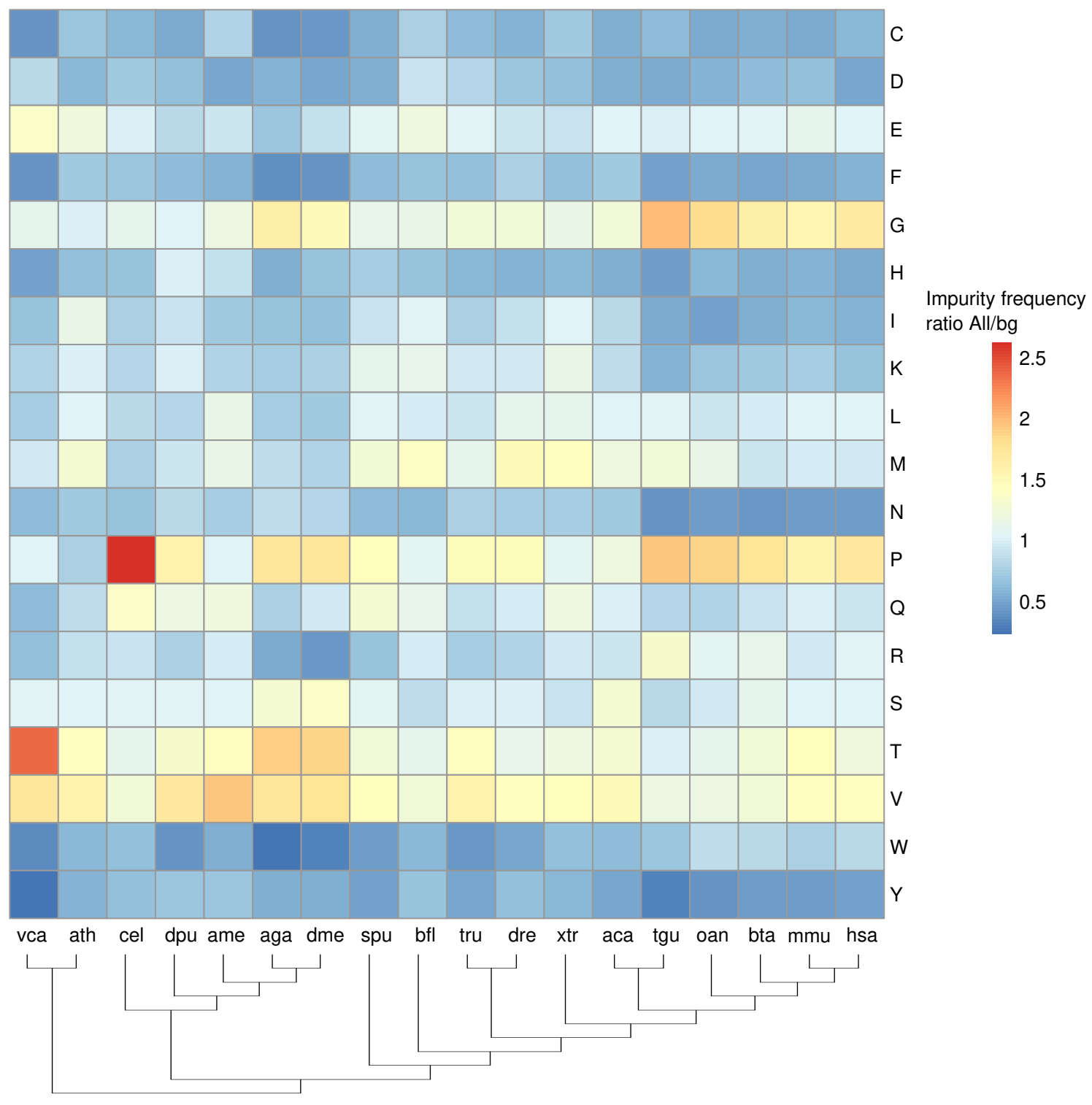

Supplement: btac610_Supplementary_Data [file btac610_supplementary_data.zip › SupplFig1.pdf]

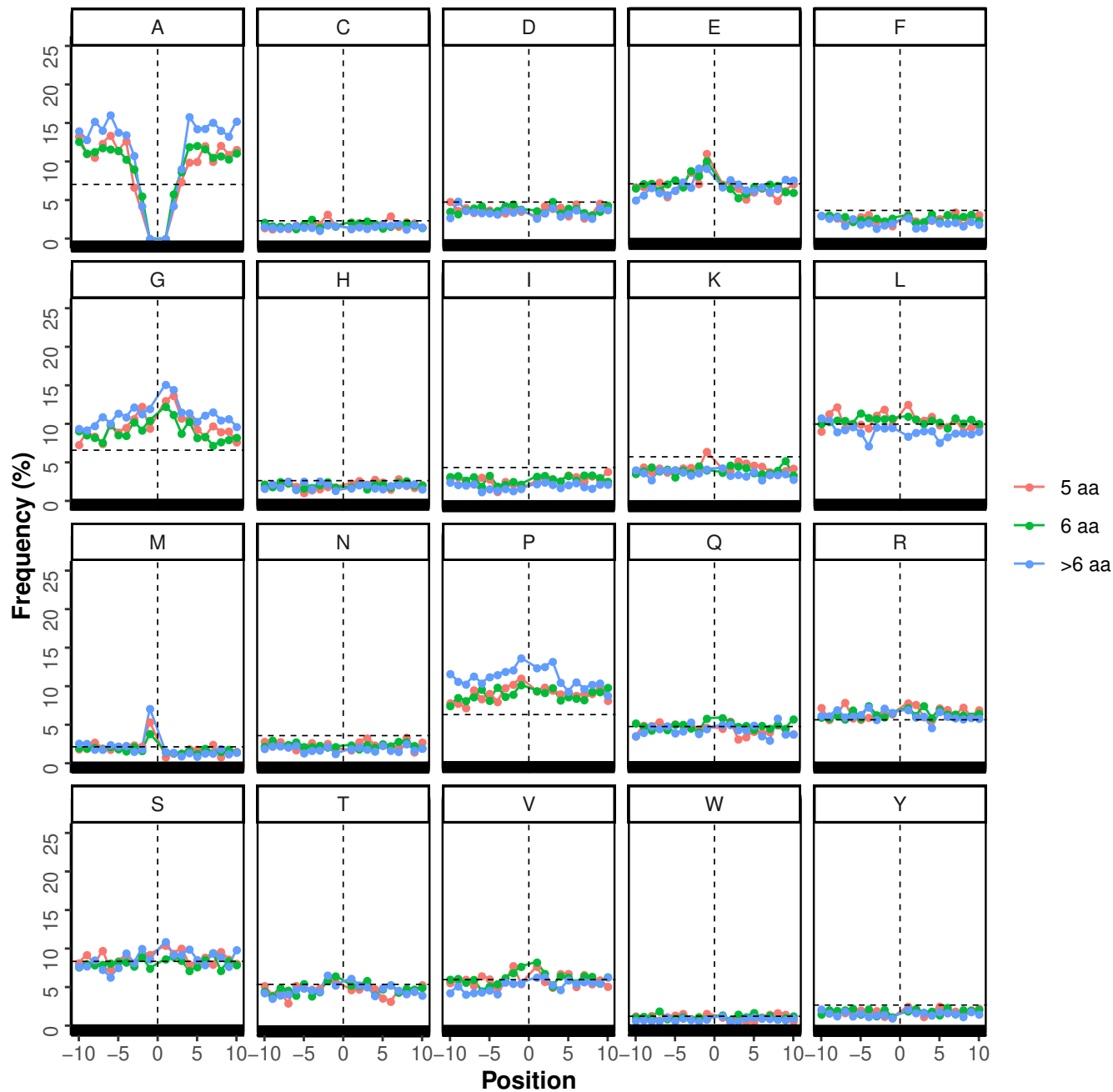

Supplement: btac610_Supplementary_Data [file btac610_supplementary_data.zip › SupplFig2.pdf]
